# Identification of Genes Involved in Antifungal Activity of *Burkholderia seminalis* Against *Rhizoctonia solani* Using Tn5 Transposon Mutation Method

**DOI:** 10.3390/pathogens9100797

**Published:** 2020-09-27

**Authors:** Muchen Zhang, Xiaoxuan Wang, Temoor Ahmed, Mengju Liu, Zhifeng Wu, Jinyan Luo, Ye Tian, Hubiao Jiang, Yanli Wang, Guochang Sun, Bin Li

**Affiliations:** 1 State Key Laboratory of Rice Biology and Ministry of Agriculture Key Laboratory of Molecular Biology of Crop Pathogens and Insects, Institute of Biotechnology, Zhejiang University, Hangzhou 310058, China; 11816060@zju.edu.cn (M.Z.); 11516057@zju.edu.cn (X.W.); temoorahmed@zju.edu.cn (T.A.); 11716062@zju.edu.cn (M.L.); 21916082@zju.edu.cn (Z.W.); 11916080@zju.edu.cn (Y.T.); 371112@zju.edu.cn (H.J.); 2 Department of Plant Quarantine, Shanghai Extension and Service Center of Agriculture Technology, Shanghai 201103, China; toyanzi@126.com; 3 State Key Laboratory for Managing Biotic and Chemical Threats to the Quality and Safety of Agro-products, Zhejiang Academy of Agricultural Sciences, Hangzhou 310021, China; sungc@mail.zaas.ac.cn

**Keywords:** antagonism, Tn5, *Burkholderia seminalis*, *Rhizoctonia solani*, rice sheath blight

## Abstract

*Rhizoctonia solani* is the causative agent of rice sheath blight disease. In a previous study, we found that the growth of *R. solani* was inhibited by *Burkholderia seminalis* strain R456. Therefore, the present study was conducted to identify the genes involved in the antifungal activity of *B. seminalis* strain R456 by using a Tn5 transposon mutation method. Firstly, we constructed a random insertion transposon library of 997 mutants, out of which 11 mutants showed the defective antifungal activity against *R. solani*. Furthermore, the 10 antagonism-related genes were successfully identified based on analysis of the Tn5 transposon insertion site. Indeed, this result indicated that three mutants were inserted on an indigenous plasmid in which the same insertion site was observed in two mutants. In addition, the remaining eight mutants were inserted on different genes encoding glycosyl transferase, histone H1, nonribosomal peptide synthetase, methyltransferase, MnmG, sulfate export transporter, catalase/peroxidase HPI and CysD, respectively. Compared to the wild type, the 11 mutants showed a differential effect in bacteriological characteristics such as cell growth, biofilm formation and response to H_2_O_2_ stress, revealing the complexity of action mode of these antagonism-related genes. However, a significant reduction of cell motility was observed in the 11 mutants compared to the wild type. Therefore, it can be inferred that the antifungal mechanism of the 10 above-mentioned genes may be, at least partially, due to the weakness of cell motility. Overall, the result of this study will be helpful for us to understand the biocontrol mechanism of this bacterium.

## 1. Introduction

*Rhizoctonia solani* is an important rice pathogenic fungus, which causes rice sheath blight (ShB) disease that may lead to a global food crisis [1]. Numerous efforts, in particular the use of chemical fungicides, have been made to control this disease. However, some concerns have been raised about the effect of synthetic chemicals on human health and the environment as well as pathogen resistance due to the long-term use of chemical fungicide in food crops [2,3]. In the past few years, the biological control of ShB was also reported in several studies by using various microbial antagonists such as *Bacillus subtilis* strain 916 [4], *Chaetomium aureum* strain MF-91 [5], *Trichoderma asperellum* [6] and *Phylloplane* yeasts [7].

Our previous studies revealed that *Burkholderia seminalis* strain R456, that was isolated from rice rhizosphere soils in vitro, inhibited the mycelial growth of *R. solani* and significantly reduced disease incidence under greenhouse conditions [8]. However, no significant inhibition of the mycelial growth was observed by the other three strains of this *Burkholderia* species [9]. It is well known that the gram-negative *B. seminalis* belongs to the *Burkholderia cepacia* complex [10] and widely exists in soil, water and plants. The inhibitory activity of *B. seminalis* strain R456 against *R. solani* may be mainly due to the production of metabolites [11].

It is essential to elucidate which genes are involved in the antagonism of strain R456, which is usually studied by constructing either overexpression or knockout deletion mutants [12]. However, the two methods are challenging for studies of unknown genes. Fortunately, an in vivo transposition system, the EZ-Tn5 Transposome (Epicentre, USA), has been shown to be an efficient and reliable method for the construction of mutant libraries in many different microorganisms by random insertion of transposon DNA into bacterial genome [13]. Furthermore, this modified EZ-TN5 system is superior to other transposon systems in consistently delivering only a single copy per chromosome [14]. Indeed, using this technique, we have not only constructed the mutant library, but also have identified the virulence-related genes from the watermelon fruit blotch pathogen *Acidovorax citrilli* by evaluating the pathogenicity of each mutant to seedlings [15].

The objective of the present study was to identify and characterize the genes involved in the antifungal activity of *B. seminalis* strain R456 against *R. solani* by constructing a Tn5 transposon insertion mutation library, identifying the target genes by inverse polymerase chain reaction (PCR) and comparing the difference between wild type and mutants.

## 2. Results and Discussion

### 2.1. Construction of Tn5 Mutant Library

**Figure 1:**
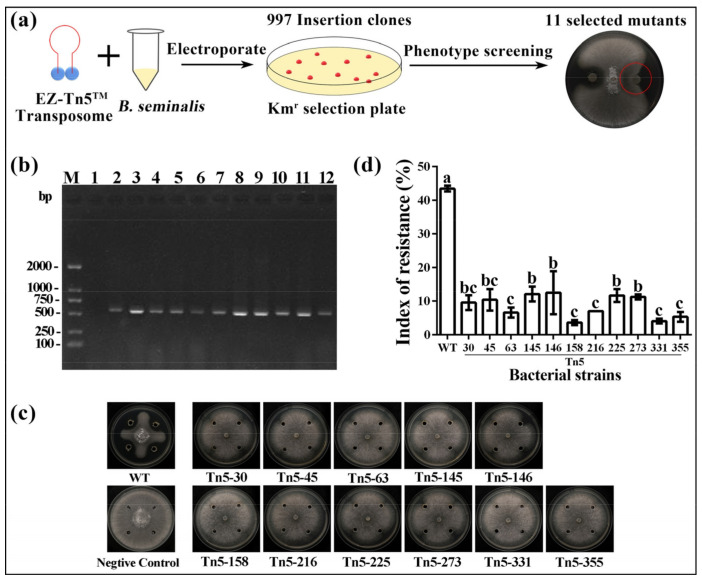
(**a**) Flow diagram of constructing Tn5 mutant library. (**b**) Gel electrophoresis of polymerase chain reaction (PCR) products of Km^r^ gene from the kanamycin-resistant colonies. M: 2000 bp DNA marker; 1: wild type; 2–12: Tn5-30, -45, -63, -145, -146, -158, -216, -225, -273, -331, -355. (**c**) Antagonistic effect of the 11 Tn5 transposon mutants and the wild type of *B. seminalis* strain R456 against *R. solani*. (**d**) Index of resistance (IR) of the 11 Tn5 transposon mutants. IR was determined as described in Section 3.3. Data were presented as means ± standard errors (n = 3). Columns with different letters (a–c) are significantly different according to LSD test (*p* < 0.05).

A comprehensive transposon mutant library (designated as Tn5-1…Tn5-997) was successfully constructed in this study based on random Tn5-inserted mutagenesis, which generated 997 kanamycin-resistant mutants (Figure 1a). This provided a foundation for us to study the function of large numbers of genes. According to the result of Dawoud et al. (2014), about 25,000 mutants would be necessary to comprehensively screen the complete genome of strain R456, which consists of 7170 genes [16]. Due to the existence of Km^r^ gene on EZ-Tn5, but not in the wild type, the successful insertion of Tn5 transposon into the genome confers Km resistance on the mutants, and the Tn5 insertion could be further identified by detecting the Km^r^ gene of Tn5 mutants. Therefore, Tn5 mutants were further verified by a single band with a size of 567 bp following PCR amplification using self-designed Tn5 primers Kan-F and Kan-R (Figure 1b).

Results of plate confrontation indicated that 11 mutants from the Tn5 transposon mutant library of strain R456 showed reduced inhibition of the growth of *R. solani* on potato dextrose agar (PDA) medium compared to the wild type (Figure 1c). In general, the mutant screen hit rate was 1.1% in this study, which was slightly higher than that (about 0.5%) of other genetic screens in *Burkholderia* biocontrol agents [17]. The resistance index of the 11 Tn5 mutants varied from 3.5–12.5%, while the resistance index of the wild type was 43.0%, suggesting that the 11 mutants significantly reduced, or even lost, the antagonistic activity against *R. solani* compared to the wild type. Furthermore, a difference was observed in the resistance index among the 11 mutants, indicating that these genes play a substantial but differential role in inhibiting the growth of *R. solani* (Figure 1d). In addition, the antagonistic effect was unaffected by the other 986 mutants compared to the wild type.

### 2.2. Location of Tn5 Inserted Sites

**Figure 2:**
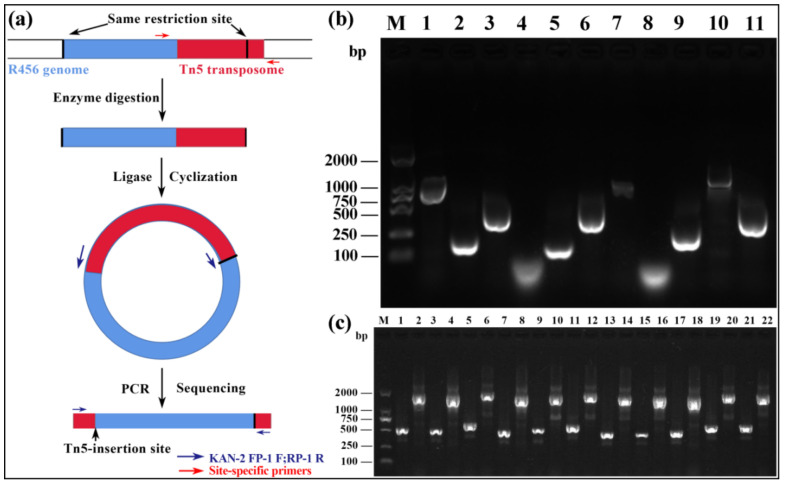
(**a**) Flow diagram of inverse PCR. (**b**) Gel electrophoresis of inverse PCR products from Tn5 transposon mutants using specific primers KAN-2 FP-1 F and KAN-2 RP-1 R. M: 2000 bp DNA marker; 1–11: Tn5-30, -45, -63, -145, -146, -158, -216, -225, -273, -331, -355. (**c**) Verification of Tn5 insertion site by using site-specific primers. M: 2000 bp DNA marker; 1–22: wild type (1, 3, 5, 7, 9, 11, 13, 15, 17, 19 and 21) and Tn5 transposon mutants (2, 4, 6, 8, 10, 12, 14, 16, 18, 20 and 22) of Tn5-30, -45, -63, -145, -146, -158, -216, -225, -273, -331, -355, respectively.

The Tn5 transposon insertion sites were determined by inverse PCR of the genomic DNAs (Figure 2a), which were extracted from the 11 selected mutants using specific primers KAN-2 FP-1 F and KAN-2 RP-1 R provided by the EZ-Tn5™ <KAN-2>Tnp Transposome™ Kit (Supplementary Table S1). Furthermore, gel electrophoresis analysis of inverse PCR products indicated that each mutant produced a single band with a size ranging from 55 bp to 1173 bp, which at least partially verified the single Tn5 insertion (Figure 2b). The Tn5 inserted sites were determined by aligning the flanking sequences with strain R456 genome and Tn5 transposon sequence, respectively, which showed 99.3–100% homology with the corresponding genes. Indeed, 10 different insertion sites were identified from the 11 mutants due to two mutants having the same transposon insertion sites in the indigenous plasmid. In addition, the Tn5 transposon insertion sites were further verified by using site-specific primers (Supplementary Table S1), which were designed based on the nucleotide sequence of each gene. As shown in Figure 2c, the amplified fragments of all mutants were 1221 bp (Tn5 transposon size) larger than those of the corresponding wild type, which further proved the accuracy of gene localization.

### 2.3. Functional Prediction of Antagonism-Related Genes

Table 1 presents the putative functions of the 10 antagonism-related genes, which were predicted based on the website of NCBI BLAST. Tn5-30 represents the insertion mutation in glycosyl transferase, which is involved in synthesis of many antifungal metabolites such as lipopolysaccharide and occidiofungin [18,19]. Tn5-45 represents the insertion mutation in histone H1, which has been regarded as an antimicrobial peptide, an endogenous natural small molecular polypeptide with broad-spectrum antibacterial and antifungal activities [20]. Tn5-63 represents the insertion mutation in nonribosomal peptide synthetase, which plays an important regulatory role in metabolism by synthesizing siderophore [21]. Tn5-145 represents the insertion mutation in 50S ribosomal protein L11 methyltransferase PrmA, which was predicted to be responsible for the methylation of ribosomal protein L11 [22]. Tn5-216 represents the insertion mutation in FAD-binding protein MnmG, whose homologous GidA can control the expression of quorum sensing genes in biocontrol bacteria [23]. Tn5-225 represents the insertion mutation in the YeiH family putative sulfate export transporter. Tn5-273 represents the insertion mutation in catalase/peroxidase HPI, which was predicted to be important for detoxifying ROS in bacteria [24,25,26]. Tn5-331 represents the insertion mutation in sulfate adenylyl-transferase subunit CysD, which was predicted to be the precursor of many metabolites with important biological functions [27]. In agreement with the results of our study, previous research has shown that the possible mechanisms of antifungal activity include production of antifungal metabolites and competition for space and nutrients [2]. Furthermore, biofilm formation, quorum sensing and alleviation of oxidative damage have also been found to be involved in suppression of fungal pathogens [28].

Interestingly, the Tn5 transposon insertion sites of the remaining three mutants were located in an indigenous plasmid. Indeed, Tn5-146 represents the insertion mutation in the membrane integrity-associated transporter subunit PqiC, which was predicted to be important for membrane integrity [29]. Tn5-158 and Tn5-355 that were inserted in the same site, represent an insertion mutation in a hypothetical protein. Thus, only mutant Tn5-158 was selected for further analysis. Blast analysis indicated that the plasmid sequences had high homology to that of *Burkholderia cenocepacia* (98.10%), *Burkholderia metallica* (93.76%) and *Burkholderia lata* (96.85%). Interestingly, *B. cenocepacia* has been reported to have antagonistic effect against the fungal pathogen *R. solani* [30]. It should also be noted that only strain R456 has this plasmid compared with the other strains of *B. seminalis*, which showed no antifungal activity against *R. solani* [9], indicating that the indigenous plasmid may be associated with the antagonistic effect of strain R456 against *R. solani*. In agreement with the results of this study, Coplin et al. (1989) reported that the plasmid was able to encode an antagonistic protein [31]. In addition, it has been reported that with the help of the plasmid, bacteria were able to obtain new phenotypes such as antibiotic resistance, pathogenicity and metabolic capacity [32,33].

### 2.4. Verification by Constructing Deletion Mutants and Complements

**Figure 3:**
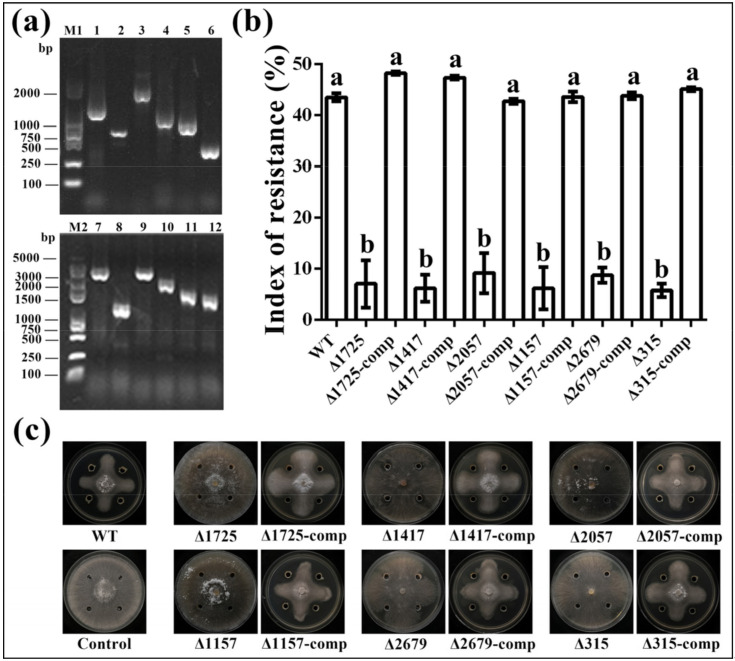
(**a**) PCR verification of six antagonism-related genes of *B. seminalis* strain R456 using target gene primers. M1: 2000 bp DNA marker; 1–6: the deletion mutants of genes Δ1725, Δ1417, Δ2057, Δ1157, Δ2679 and Δ315; 7–12: the complements of the deletion mutants in 1–6. (**b**) Index of resistance of the six genes deletion mutants and complements. The index of resistance was determined as described in Section 3.3. Data were presented as means ± standard errors (n = 3). Columns with different letters (a, b) are significantly different according to LSD test (*p* < 0.05). (**c**) Antagonistic effect of the six genes deletion mutants and complements against *R. solani*.

To further verify the reliability of the Tn5 insertion mutation, we constructed deletion mutants and the corresponding complements of the 6 antagonism-related genes related to Tn5-30, Tn5-45, Tn5-63, Tn5-216, Tn5-225 and Tn5-331. Following the screening of Km^r^ resistance, the constructed mutants of the above-mentioned genes were verified by PCR amplification using target gene primers, which produced a single gel band with the expected sizes of about 1274, 577, 1688, 1009, 822 and 400 bp, respectively (Figure 3a). Furthermore, the resulting mutants were successfully complemented in this study, which was confirmed by producing a single gel band with the expected sizes of 4240, 1158, 3900, 2481, 1700 and 1521 bp, respectively (Figure 3a). In addition, bacterial identity was determined by the analysis of 16S rDNA sequences of the constructed mutants and complements, which showed the highest homology to *B. seminalis* with 98–100% similarity by blasting against the NCBI database (data not shown).

The constructed mutants and complements were further tested for their antifungal activity against *R. solani*. As showed in Figure 3c, antifungal activity was significantly reduced in the five mutants compared to the wild type, while the index of resistance (IR) of the deletion mutants of the six genesΔ1725, Δ1417, Δ2057, Δ1157, Δ2679 and Δ315 was 7.00%, 6.16%, 9.13%, 6.16%, 8.70% and 5.73%, respectively (Figure 3b). However, there was no significant difference in antifungal activity between the wild type and the six complemented strains, which had index of resistance (IR) values of 48.24%, 47.32%, 42.74%, 43.56%, 43.78%, and 45.09%, respectively (Figure 3b). This result of the study not only revealed that these genes were associated with the antifungal activity of strain R456 against *R. solani*, but also verified the reliability of the EZ-Tn5 transposition system.

### 2.5. Growth of Antagonism-Related Tn5 Transposon Mutants

Results from this study indicated that the cell numbers of the wild type increased continuously, while the OD600 values were 0.50, 0.90, 1.12 and 1.31, respectively, at 6, 12, 18 and 24 h of incubation. Similar trends were observed in the growth of the 10 Tn5 mutants of strain R456. However, this result also clearly indicated that the mutation of Tn5 caused a differential effect in the growth of strain R456. For example, growth was significantly inhibited by the mutations of Tn5-63, Tn5-30 and Tn5-216, which caused a 20.0%, 32.0% and 40.0% reduction in the OD600 values compared to the wild type, but growth was almost unaffected by the other seven mutants after 6 h of incubation. Furthermore, growth was significantly inhibited by Tn5-63 and Tn5-216, with a 10.0% and 18.9% reduction in OD600 values, respectively. Growth was significantly promoted by Tn5-145, Tn5-146, Tn5-158 and Tn5-225, with a 17.8%, 10.0%, 10.0% and 8.9% increase in OD600 values, respectively. No difference was found in OD600 values between the other four Tn5 mutants and the wild type after 12 h of incubation. Growth was significantly inhibited by Tn5-225 and Tn5-216, with a 6.3% and 19.6% reduction in OD600 values, respectively. Growth was significantly promoted by Tn5-145, with a 6.3% increase in OD600 value. No significant difference was found in the OD600 values between the other eight mutants and the wild type after 18 h of incubation. In addition, growth was significantly inhibited by Tn5-216 and Tn5-225, with a 16.0% and 20.6% reduction in the OD600 values, respectively, while the growth was unaffected by the other eight mutants compared to the wild type after 24 h of incubation (Table 2).

Obviously, the influence of Tn5 mutation on bacterial growth depended on the gene and incubation time. Much research has shown that the reduction in the inhibitory effect of antagonistic bacteria by gene mutation may be main due to disturbance in cell growth [34]. In contrast, the Tn5 mutants constructed in this study showed an increase, decrease or no change in bacterial growth compared to the wild type of strain R456, indicating that the reduced antifungal activity in the mutants may be due to factors other than growth disturbance. However, it was also noted that the growth of Tn5-216 was significantly inhibited at the four tested incubation times. Thus, it can be inferred that the reduced antifungal activity of this transposon mutant against *R. solani* may be, at least partially, due to poor bacterial growth. These results revealed the complexity of the antagonistic mechanism in strain R456.

### 2.6. Biofilm Formation

**Figure 4:**
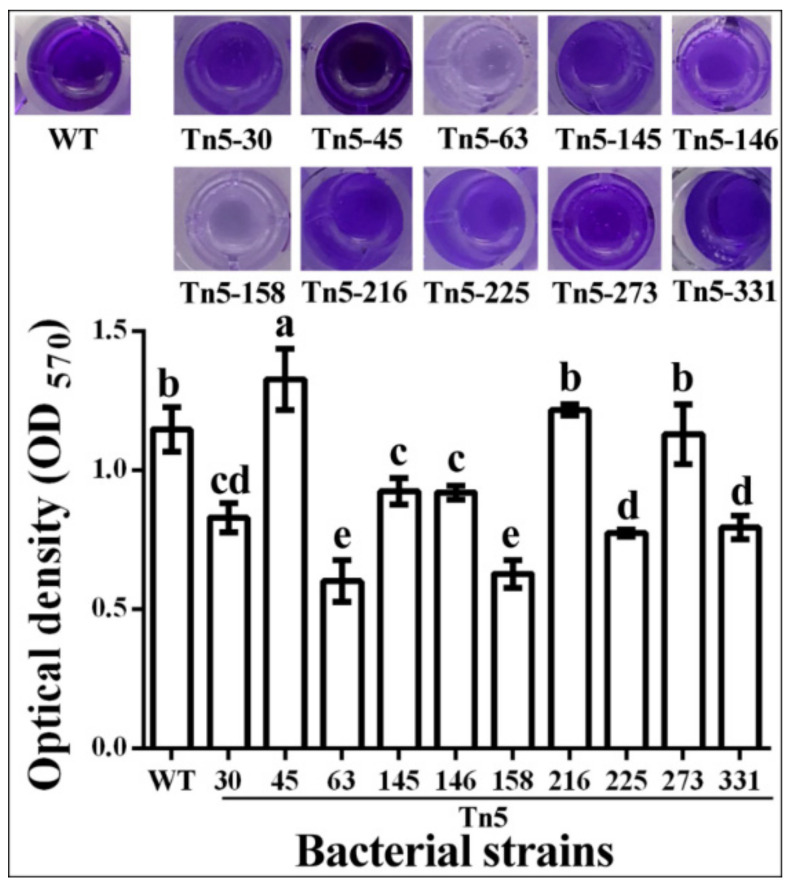
Biofilm forming ability of Tn5 transposon mutants and the wild type. Optical density at 570 nm (OD570) is presented as means ± standard errors (n = 3). Columns with different letters (a–e) are significantly different according to LSD test (*p* = 0.05). WT: wild-type.

The results of this study showed that mutation of the 10 antagonism-related genes caused differential effects on biofilm formation, which were determined based on the method of crystal violet staining by measuring the OD570 value. Indeed, biofilm formation was unaffected by Tn5-216 and Tn5-273. However, biofilm formation was significantly reduced by Tn5-30, Tn5-63, Tn5-145, Tn5-146, Tn5-158, Tn5-225 and Tn5-331, which caused a 27.7%, 47.5%, 19.4%, 19.8%, 45.3%, 32.5% and 30.7% reduction, respectively, in the OD570 value. In contrast, biofilm formation was significantly increased by Tn5-45, which caused a 15.7% increase in the OD570 value compared to the wild type (Figure 4).

It has been well known that biofilm formation is a dynamic and complex process involving signal transduction systems, transcriptional regulation, and stress response, which make it important for both beneficial bacteria and plant pathogenic bacteria [15,35]. In agreement with the results from some other studies, most of the mutants significantly reduced biofilm formation, which may, at least partially, be attributed to the reduced antifungal activity against *R. solani*. However, this study also found that there was no, or even an increased, effect on biofilm formation of several mutants, suggesting that biofilm formation may not be the leading cause for the antifungal mechanism of strain R456 against *R. solani*.

### 2.7. Motility

**Figure 5:**
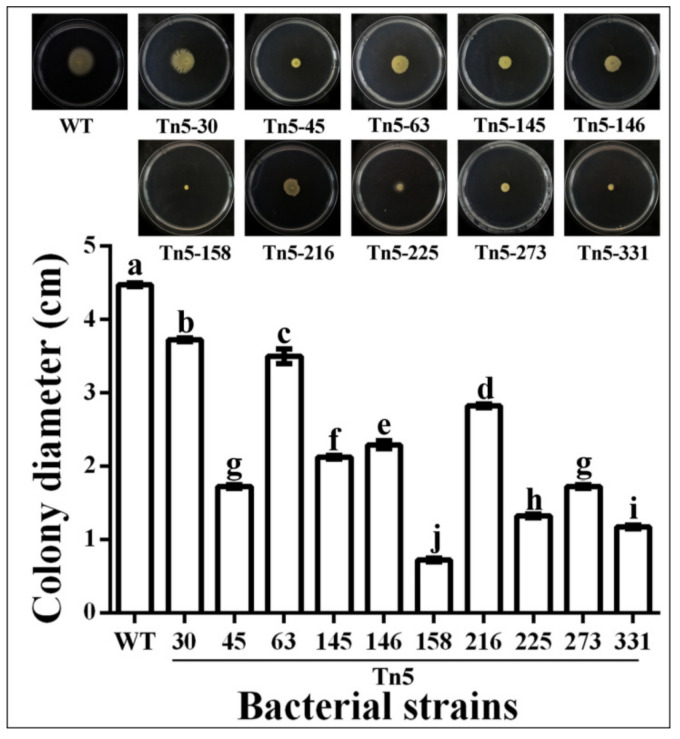
Motility of Tn5 transposon mutants and the wild type of strain R456. Colony diameter (cm) is presented as means ± standard errors (n = 3). Columns with different letters (a–j) are significantly different according to LSD test (*p* = 0.05). WT: wild-type.

Motility is a basic physiological phenotype of bacteria, which contributes to bacterial colonization, survival, virulence and antagonism [36]. The influence of gene mutation on motility was determined in this study by adding 10 μL of bacterial suspension with an OD600 value of 1.0 in the center of a semisolid plate and then measuring colony diameter. As shown in Figure 5, motility was significantly reduced by all of Tn5 mutants after 48 h of incubation. Indeed, the colony diameter of the wild type was 4.47 cm, while there was a 16.8%, 61.5%, 21.8%, 52.5%, 48.7%, 36.9%, 70.4%, 61.5%, 73.7% and 88.0% reduction in the colony diameter of Tn5-30, Tn5-45, Tn5-63, Tn5-145, Tn5-146, Tn5-216, Tn5-225, Tn5-273, Tn5-281 and Tn5-158, respectively, compared with the wild-type. The result of this study clearly revealed that the reduced antifungal effect of Tn5 mutants against *R. solani* may be, at least partially, due to their weak motility. A potential association was also observed between motility and production of antifungal metabolites. In agreement with the results of this study, it has been well known that adaptive motility is intrinsically associated with bacterial metabolism [37,38,39]. Therefore, it can be inferred that the weakness of motility in 10 mutants may be due to a change in metabolites.

### 2.8. Tolerance to Hydrogen Peroxide (H_2_O_2_)

**Figure 6:**
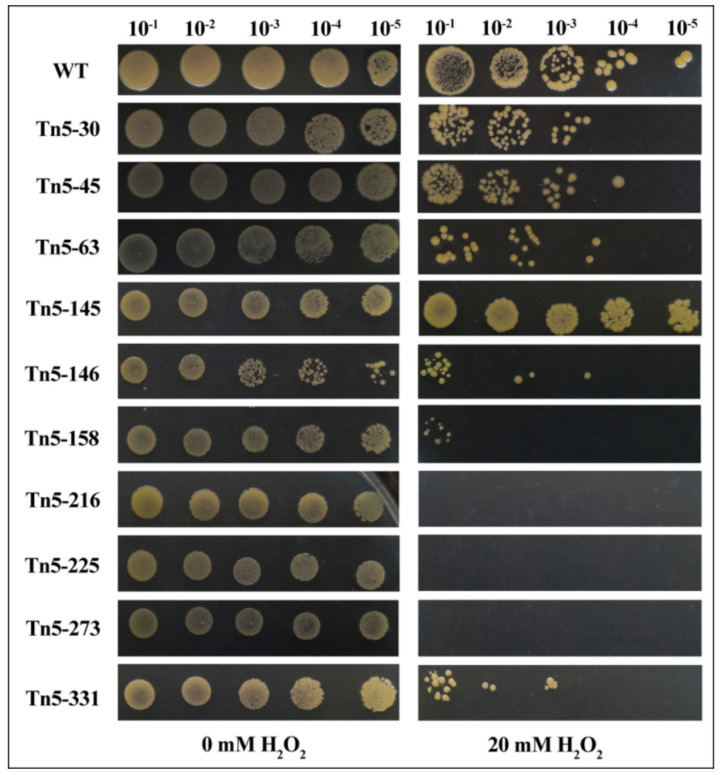
Tolerance of Tn5 transposon mutants and wild type to 20 mM H_2_O_2_ of H_2_O_2_. Bacterial tolerance to H_2_O_2_ was determined by mixing the 10-fold serial dilutions (10^−1^ to 10^−5^) of bacterial suspensions (OD600 = 0.5) with H_2_O_2_ and then counting the number of colonies on LB medium following the incubation at 30 °C for 24 h.

Studies have shown that the activity level of catalase is one of the mechanisms contributing to biological control activity [25]. Therefore, the influence of gene mutation on catalase activity was determined by measuring their response to H_2_O_2_. As shown in Figure 6, no significant difference was seen in H_2_O_2_ tolerance between Tn5-145 and the wild type. However, the tolerance of Tn5-216, Tn5-225, Tn5-273 to H_2_O_2_ was reduced by five orders of magnitude, the tolerance of Tn5-158 to H_2_O_2_ was decreased by four orders of magnitude, the tolerance of Tn5-30, Tn5-63, Tn5-146, Tn5-331 and Tn5-355 to H_2_O_2_ was decreased by two orders of magnitude, and the tolerance of Tn5-45 to H_2_O_2_ was decreased by one order of magnitude. This result indicated that these genes differentially affected the activity of catalase.

Tolerance to H_2_O_2_ reflects the adaptability of bacteria to oxidative stress, which is important for their survival under unfavorable conditions [40]. In general, the results of this study indicated that mutation of the 10 antagonism-related genes caused a differential effect in H_2_O_2_ tolerance. However, most of the Tn5 mutants in the study reduced H_2_O_2_ tolerance, indicating the potential association of antifungal activity with tolerance to H_2_O_2_. Among them, we paid more attention to Tn5-273, in which the Tn5 transposon was inserted in catalase/peroxidase HPI, which may be the main cause for the reduction in the tolerance of Tn5-273 to H_2_O_2_. On the other hand, the change in H_2_O_2_ tolerance was also achieved for other mutants, which may be due mainly to a pleiotropic effect of these genes.

## 3. Materials and Methods

### 3.1. Bacterial Strains and Growth Conditions

In our previous study, *B. seminalis* strain R456 was isolated from the rice rhizosphere soil, which has strong antagonistic activity against *R. solani* [8,9]. *B. seminalis* strains were cultured in Luria-Bertani (LB) agar or broth medium (Oxoid Ltd., Basingstoke, Hants, UK) [41] at 30 °C, while *Escherichia coli* strains were grown in LB agar or broth medium at 37 °C. The fungal pathogen *R. solani* was isolated from diseased rice plants in Zhejiang province, China and incubated on potato dextrose agar (PDA; Oxoid Ltd.) at 28 °C [8]. When required, the culture media were supplied with the antibiotics kanamycin (Km), ampicillin (Amp) and chloramphenicol (Chl) with a final concentration of 200, 50 and 3.4 μg/mL, respectively. All antibiotics were purchased from Sangon Biotech Co., Ltd., Shanghai, China.

### 3.2. Transposon Insertion of Tn5

A library of Tn5 transposon mutants of *B. seminalis* R456 was constructed using the MA138E-EZ-Tn5-KAN-2 TNP Transposome Kit, which was carried out according to the manufacturer’s protocol (Epicentre Biotechnologies, Madison, WI, USA). The competent cells and electroporation transformation were prepared using a previously described method [15] with slight modifications. In brief, following precooling for 40 min, bacterial cells were suspended in 40 μL sterile water to obtain a final concentration of 1 × 10^10^ CFU/mL, and then added to 1 μL of the EZ-Tn5 transposon (Epicentre Biotechnologies, Madison, WI, USA). The highest transformation efficiencies were achieved by electrotransformation at 2200 V, 25 μF and 400 Ω. The gap width for electroporation was 2 mm. Tn5 mutants were determined based on their growth on LB agar containing kanamycin (200 μg/mL) and PCR amplifications of genomic DNAs from different colonies, which were carried out using the specific primer pairs Kan-F and Kan-R, which were self-designed according to the sequence of the Km^r^ gene on EZ-Tn5 (Supplementary Table S1). The procedure for the PCR reaction was as follows: one cycle for predenaturation at 94 °C, 5 min; 35 cycles for denaturation at 94 °C, 30 s; annealing at 62 °C, 30 s; extension at 72 °C, 1 min; and one cycle for extension at 72 °C, 7 min.

### 3.3. Evaluation of Antagonistic Activity of Tn5 Mutants

Antagonistic activity of all the constructed Tn5 mutants against *R. solani* was determined based on the plate confrontation method, which was carried out as described by Balouiri et al. (2016) [42]. Briefly, the Tn5 mutants were evenly distributed around the mycelial plug of *R. solani*, which was inoculated in the center of a PDA medium plate and then cultured at 28 °C until the negative control *R. solani* grew to full plate. The antagonistic activity was evaluated by the Index of Resistance (IR), which was calculated using the following formula:

$$\mathrm{IR}\;(\%)\;=\frac{\left(D-6)-(d-6\right)}{\left(D-6\right)}\times 100$$

where D is the mycelial diameter of *R. solani* that grew in the PDA plate as the negative control, d is the mycelial diameter of *R. solani* in the presence of *B. seminalis*, and 6 (mm) is the diameter of the inoculated mycelial plug of *R. solani*.

Further studies were carried out on the Tn5 mutants, which caused a significant change in the antagonistic activity against *R. solani* compared to the wild type of *B. seminalis* strain R456.

### 3.4. Identifying the Location of Transposon Insertions

Following the extraction of genomes DNAs from the Tn5 mutants, digestion by restriction endonuclease *Sal*I (or *Pae*I), and ligation using T4 DNA ligase, the transposon insertion sites were determined by inverse PCR using the primers KAN-2 FP-1 F and KAN-2RP-1 R provided by the EZ-Tn5™ <KAN-2>Tnp Transposome™ Kit (Epicentre Biotechnologies, Madison, WI, USA) (Supplementary Table S1). The procedure of the inverse PCR reaction was as follows: one cycle for predenaturation at 94 °C, 5 min; 35 cycles for denaturation at 94 °C, 30 s; annealing at 58 °C, 30 s; extension at 72 °C for 1 min; and one cycle for extension at 72 °C, 7 min. The PCR amplified products were analyzed on 1% agarose gels and then sequenced by TsingKe Biological Technology (Hangzhou, China). The Tn5 insertion sites were identified by aligning the obtained flanking sequences with strain R456 genome (Accession number: PRJNA76131) and the Tn5 transposon sequence, respectively, while the function of the genes disrupted by Tn5 insertion was predicted based on the website of NCBI BLAST (https://blast.ncbi.nlm.nih.gov/Blast.cgi). In addition, the Tn5 transposon insertion were justified by designing site-specific primers pairs (Supplementary Table S1) to amplify each specific gene in Tn5 mutants and the wild type, in which the former was 1221 bp larger in size than that of latter due to the insertion of Tn5.

### 3.5. Generation of Deletion Mutants and Complemented Strains

To further validate the role of antagonism-related genes in the antifungal activity of strain R456, an insertional mutagenesis on the target gene was generated by suicide plasmid pJP5603 through homologous recombination on the background of wild type strain R456. Construction and complementation of in-frame gene deletions were determined by culturing the bacteria at 30 °C, 200 rpm/min in LB broth supplemented with 50 μg/mL kanamycin (Km) or chloromycetin (Chl), respectively, which was carried out as described by Liu et al. (2012) [43]. Following the screening of Km and Chl + Km resistance, respectively, the constructed Tn5 mutants and the complemented strains were verified by PCR amplification using different primers, which are shown in Supplementary Table S1. In addition, to rule out possible contamination, we carried out the sequence analysis of 16S rDNA of Tn5 mutants and the complemented strains, which were amplified as described by Ogunyemi et al. (2016) [44].

### 3.6. Growth Measurement

Bacterial growth was determined according to the method of [15] by measuring the OD600 values of the wild type and Tn5 insertion mutants of strain R456 using a microplate spectrophotometer (Thermo Fisher Scientific Inc., Waltham, MA, USA). In brief, after inoculating 50 μL of overnight-cultured bacterial suspension (OD600 = 1.0) into 5 mL of LB broth, the mixtures were then incubated at 30 °C, 200 rpm/min for 6, 12, 18 and 24 h, respectively. Each treatment had three replicates and the experiment was repeated twice.

### 3.7. Biofilm Formation Ability

Biofilm formation assays were performed in 96-well polystyrene culture plates using a crystal violet stain described previously [45] and were carried out by measuring absorbance at 570 nm using a microplate spectrophotometer (Thermo Fisher Scientific Inc., Waltham, MA, USA). In brief, each well in the 96-well microplates was inoculated with 200 µL of bacterial suspension with a final concentration of approximately 1 × 10^6^ cells/mL. After 16 h of static incubation at 30 °C, the medium was decanted gently, and the wells were washed with sterile water to remove the cells floating on the surface and the loosely attached cells. Adherence was monitored by staining the remaining firmly attached cells in biofilm with 1% crystal violet at room temperature for 10 min. Excess crystal violet was removed by washing the wells with sterile water. The attached cells’ bound crystal violet was solubilized with 1 mL of 90% ethanol and quantified.

### 3.8. Motility

Cell motility of Tn5 mutants and the wild type of strain R456 was determined using a previously described method [44,46]. In brief, bacteria were cultured in LB broth at 30 °C, 200 rpm/min. After centrifugation and resuspension with sterile water, the final concentration of bacterial suspension was adjusted to 10^5^ CFU/mL. Colony diameter was measured by inoculating 5 μL of cell suspension onto the center of the semisolid LB medium containing 0.3% agar and then incubating the plate at 30 °C for 48 h. The experiment was repeated three times independently, and the colony photographed and analyzed.

### 3.9. Tolerance to H_2_O_2_

Bacterial tolerance to H_2_O_2_ was determined by counting the number of colonies using a previously described method with minor modifications [47,48]. In brief, bacteria were cultured overnight in LB broth at 30 °C, 220 rpm/min, while the final concentration was adjusted to 0.5 of OD600. Then, bacterial suspensions were 10-fold serially diluted to 10^−5^ with sterile 0.9% saline. After mixing the serial dilutions of the bacterial suspensions with H_2_O_2_ with a final concentration of 20 mM, the mixture was spotted on LB medium and then incubated at 30 °C for 24 h. Negative controls were prepared using serial dilutions of the bacterial suspensions alone.

### 3.10. Statistical Analyses

The software IBM SPSS Statistics, version 19 was used to perform the statistical analyses. Levels of significance (*p* < 0.05) of the main treatments and their interactions were calculated by analysis of variance after testing for normality and variance homogeneity.

## 4. Conclusions

In the present study, we constructed a random mutant library of *B. seminalis* strain R456 by using the Tn5 transposon mutation method. Among the identified 997 mutants, 11 mutants showed reduced antifungal activity against *R. solani*. Furthermore, 10 genes involved in antifungal activity of strain R456 were identified using inverse PCR. The bacteriological test revealed that the 10 antagonism-related genes had a differential role in cell growth, biofilm formation and resistance to oxidative stress, revealing their complexity and diversity. However, motility was reduced by all 11 Tn5 mutants compared to the wild type, indicating that the antagonistic mechanism of bacterial strain R456 may be, at least partially, due to disturbance in bacterial motility. In addition, the reliability of the Tn5 transposon mutation method was verified in this study by the knockout of corresponding genes. Overall, the identification of ten antagonism-related genes will be helpful for us to further elucidate the biological control mechanisms of this bacterium.

## Figures and Tables

**Table 1:** Identification of Tn5 transposon insertion sites by inverse polymerase chain reaction (PCR) amplification and the alignment of the flanking sequences with strain R456 genome and Tn5 transposon sequence.

Mutants	Locus_Tag	Enzyme Site	Sequence Length (bp)	Function Prediction
Tn5-30	BsemR456_1725	*Sal*I	861	glycosyl transferase
Tn5-45	BsemR456_1417	*Pae*I	203	multispecies: histone H1
Tn5-63	BsemR456_2057	*Sal*I	479	nonribosomal peptide synthetase
Tn5-145	BsemR456_1423	*Sal*I	55	50S ribosomal protein L11 methyltransferase
Tn5-146	BsemR456_6327	*Sal*I	150	Plasmid, membrane integrity-associated transporter subunit PqiC
Tn5-158	BsemR456_6210	*Sal*I	452	Plasmid, hypothetical protein
Tn5-216	BsemR456_1157	*Sal*I	765	tRNA uridine-5-carboxymethylaminomethyl (34) synthesis enzyme MnmG.
Tn5-225	BsemR456_2679	*Pae*I	33	YeiH family putative sulfate export transporter
Tn5-273	BsemR456_1556	*Sal*I	281	catalase/peroxidase HPI
Tn5-331	BsemR456_315	*Sal*I	1173	sulfate adenylyltransferase subunit CysD
Tn5-355	BsemR456_6210	*Sal*I	452	Plasmid, hypothetical protein

The function of these genes was predicted based on the website of NCBI BLAST.

**Table 2:** Effect of incubation time on the growth of Tn5 mutants.

Bacterial Strains	Incubation Time (h)			
	6	12	18	24
Wild type	0.50 ± 0.03 ^ab^	0.90 ± 0.00 ^c^	1.12 ± 0.00 ^bc^	1.31 ± 0.00 ^ab^
Tn5-30	0.34 ± 0.00 ^d^	0.90 ± 0.08 ^c^	1.14 ± 0.02 ^b^	1.25 ± 0.04 ^b^
Tn5-45	0.53 ± 0.01 ^ab^	0.93 ± 0.05 ^bc^	1.09 ± 0.02 ^c^	1.27 ± 0.02 ^b^
Tn5-63	0.40 ± 0.02 ^c^	0.81 ± 0.03 ^d^	1.07 ± 0.02 ^c^	1.28 ± 0.01 ^b^
Tn5-145	0.56 ± 0.06 ^a^	1.06 ± 0.02 ^a^	1.19 ± 0.03 ^a^	1.36 ± 0.02 ^a^
Tn5-146	0.47 ± 0.08 ^b^	0.99 ± 0.04 ^ab^	1.12 ± 0.03 ^bc^	1.31 ± 0.00 ^ab^
Tn5-158	0.49 ± 0.03 ^b^	0.99 ± 0.03 ^b^	1.12 ± 0.04 ^bc^	1.28 ± 0.08 ^b^
Tn5-216	0.30 ± 0.01 ^d^	0.73 ± 0.03 ^e^	0.90 ± 0.03 ^e^	1.10 ± 0.06 ^c^
Tn5-225	0.47 ± 0.04 ^b^	0.98 ± 0.06 ^b^	1.05 ± 0.01 ^d^	1.04 ± 0.00 ^c^
Tn5-273	0.46 ± 0.03 ^bc^	0.95 ± 0.04 ^bc^	1.10 ± 0.02 ^c^	1.32 ± 0.03 ^ab^
Tn5-331	0.44 ± 0.01 ^bc^	0.93 ± 0.01 ^bc^	1.11 ± 0.03 ^bc^	1.28 ± 0.05 ^b^

Bacterial growth was measured by optical density at 600 nm (OD600). Data from repeated experiments were pooled and presented as means ± standard error. Treatments with different letters (a–e) are significantly different according to LSD test (*p* = 0.05).

## Supplementary Materials

The following are available online at https://www.mdpi.com/2076-0817/9/10/797/s1, Table S1: Primers used in this study.

## Abbreviations

BLAST	Basic local alignment search tool
IR	Index of resistance
PCR	Polymerase chain reaction
PDA	Potato dextrose agar (medium)
ShB	Rice sheath blight
WT	Wild type
