# Supplementary material for: Identification of Genes Involved in Antifungal Activity of Burkholderia seminalis Against Rhizoctonia solani Using Tn5 Transposon Mutation Method
Source: Pathogens. 2020 Sep 27;9(10):797. doi: 10.3390/pathogens9100797 (PMC7600168; doi:10.3390/pathogens9100797)
Supplement: Supplementary file 1 [file pathogens-09-00797-s001.pdf]

Supplementary Table S1. Primers used in this study.

Primers Name	Sequences (5′-3′)	Tm (℃)	CG (%)
**Identification of Tn5 mutants**			
**Kan-F**	AAGGTAGCGTTGCCAATGAT	56	45
**Kan-R**	GCCGTTTCTGTAATGAAGGA	53	45
**Amplification of unknown sequence flanking transposon Tn5**			
**KAN-2 FP-1 F**	ACCTACAACAAAGCTCTCATCAACC	58	44
**KAN-2 RP-1 R**	GCAATGTAACATCAGAGATTTTGAG	54	36
**Insertion site-specific primers**			
**30-F**	GGACGAGGTGCTCGAATTCG	59	60
**30-R**	TGGTTGCCTACCGCATCGC	61	63
**45-F**	CGCCTGATGCAAAGCATGC	59	58
**45-R**	GTGCTGCCTTCTTCGCAGC	60	63
**63-F**	GGTGGATTCTTCGCAGCGC	60	63
**63-R**	CTCTGGCCCGAAGCCAA	58	65
**145-F**	ACGGTTTCGCAATGAGGGC	59	58
**145-R**	GGCTCGAACAGTCGGTGAAG	59	60
**146-F**	TTAATCCGGGCATCGGTACT	56	50
**146-R**	CCTCGTCGCCTGTGCACTA	60	63
**158/355-F**	CGCATTGGGAGGCCTTTGAT	59	55
**158/355-R**	TTCGCCATCTGTCAATTTGACG	57	45
**216-F**	TCGATGTCCAGCTCGAGCTC	59	60
**216-R**	ACTTCAGGCGTTCGGTTTCA	58	50
**225-F**	GCTGATGTTCAGGTGTTCGGC	60	57
**225-R**	GCGATTTCCTGCAGGCTCAC	60	60
**273-F**	GGACTGGGAAGTGAACCGC	59	63
**273-R**	CGCAACTGCGAGTGCGA	60	65
**331-F**	ATAACCTTGCTTCTTGCGCTGT	58	45
**331-R**	GTCGGGCACGTCGAGGATTC	61	65
**Gene knock-out primers**			
**k30-F**	CGGGATCCTGAATCGAGACGTCTGTGTCGC	60	55
**k30-R**	CGGAATTCCCCCATGTTCATGGTCTTGAGC	59	55
**k45-F**	CGGGATCCAGGTTGCTGCCAAGAAGACC	58	55
**k45-R**	CGGAATTCGGGTTGAGCGCGGTCTTC	59	67
**k63-F**	TGCTCTAGAAATTTAACGCTGCTCCGGCT	58	50
**k63-R**	CGGAATTCTTGCGCTGCCAGCAGT	59	63
**k216-F**	CGGGATCCTATCCGCTTCCGTTCGCGT	60	58
**k216-R**	CGGAATTCTTCGGTTTCACGTGAAACAGCG	60	50
**k225-F**	CGGGATCCGTCGCGTTCTTCGGGTTGC	60	63
**k225-R**	CGGAATTCGCGGGCAATTCAGCCAAGC	61	63
**k331-F**	CGGGATCCCTGTGGAGCCTGTACAACGC	59	60
**k331-R**	CGGAATTCCTTGCTTCTTGCGCTGTTCC	58	55
**Gene complementation primers**			
**c30-F**	AGCTCGAATTCTAGACGTGTTCCCGTCCTACTACG	58	60
**c30-R**	GCAGAAGCTTCTAGATTCCTTACTCCAGCAGGCCG	60	60
**c45-F**	AGCTCGAATTCTAGAAACGCAACTTCTTCGAGACGC	59	52
**c45-R**	GCAGAAGCTTCTAGAGCGATTACGGACGGCTGC	60	67
**c63-F**	AGCTCGAATTCTAGAGGGCGCGAAATGGTGATG	58	61
**c63-R**	GCAGAAGCTTCTAGATCAGTAGGCTGCAATGTTTTCC	56	45
**c216-F**	AGCTCGAATTCTAGAGTTGGCGGGAAACGAACGG	60	63
**c216-R**	GCAGAAGCTTCTAGACGTCATTGTTGCGTCGGGAC	60	60
**c225-F**	AGCTCGAATTCTAGAGTATTGCCGCTCATCGTCTGGA	60	55
**c225-R**	GCAGAAGCTTCTAGAGCAGCTTCAGCAGGAACGAG	59	60
**c331-F**	AGCTCGAATTCTAGAACGCGGTCGACCAGTATGTC	60	60
**c331-R**	GCAGAAGCTTCTAGAAATGTCCTCGTGTGCTTCAG	56	60

Note: Nucleotides with underline indicated restriction sites of the enzymes: *Bam*HI, *Eco*RI, or *Xba*I.
